# A Single Pair of Serotonergic Neurons Counteracts Serotonergic Inhibition of Ethanol Attraction in *Drosophila*

**DOI:** 10.1371/journal.pone.0167518

**Published:** 2016-12-09

**Authors:** Li Xu, Jianzheng He, Andrea Kaiser, Nikolas Gräber, Laura Schläger, Yvonne Ritze, Henrike Scholz

**Affiliations:** 1 Zoology, Albertus Magnus University Cologne, Köln, Germany; 2 Institute of Genetics and Neurobiology, Julius Maximillian University Würzburg, Würzburg, Germany; Tohoku University, JAPAN

## Abstract

Attraction to ethanol is common in both flies and humans, but the neuromodulatory mechanisms underlying this innate attraction are not well understood. Here, we dissect the function of the key regulator of serotonin signaling—the serotonin transporter–in innate olfactory attraction to ethanol in *Drosophila melanogaster*. We generated a mutated version of the serotonin transporter that prolongs serotonin signaling in the synaptic cleft and is targeted via the Gal4 system to different sets of serotonergic neurons. We identified four serotonergic neurons that inhibit the olfactory attraction to ethanol and two additional neurons that counteract this inhibition by strengthening olfactory information. Our results reveal that compensation can occur on the circuit level and that serotonin has a bidirectional function in modulating the innate attraction to ethanol. Given the evolutionarily conserved nature of the serotonin transporter and serotonin, the bidirectional serotonergic mechanisms delineate a basic principle for how random behavior is switched into targeted approach behavior.

## Introduction

Species throughout the animal kingdom prefer ethanol-containing food sources. However, how the decision is made and is converted into to the actual food choice is not fully understood. To uncover conserved regulatory mechanisms underlying ethanol preference, we examined neuronal mechanisms behind olfactory attraction to ethanol in *Drosophila melanogaster*. *Drosophila* is a well-established genetic model system used to identify mechanisms behind human behaviors related to ethanol intake [1]. Olfactory ethanol attraction is a rather simple, innate, naturally occurring behavior [2]. Innate behaviors are robust and are elicited by an external stimulus—in this case an odor—and can be modified by the animal’s internal environment [3, 4]. The first neuroanatomical layers of olfactory information processing have been identified (reviewed by [5] and [6] and share high similarity to mammalian odor processing networks [7]. The relatively well-defined olfactory pathways and conserved molecular components underlying the regulation of innate behaviors make it possible to unravel basic principles of attraction to ethanol.

The approach to the ethanol containing food odors requires a reinforcing mechanism that involves the octopaminergic neurotransmitter system [8]. Other factors that might influence the attraction to ethanol are inhibitory mechanisms that alter the execution of the approach and therefore allowing the animal to dynamically adjust their goal oriented behaviors [9]. However these inhibitory mechanisms have not been identified for the attraction to ethanol.

Serotonin is implicated in the modulation of odor information processing in humans and insects [10, 11]. For example in *Drosophila* in the presence of an odor serotonin increases neuronal activity in second order neurons—the projection neurons—of the olfactory pathway [12]. Within the olfactory pathway serotonin is also involved in the regulation of higher brain function such as learning and memory of negative reinforced odor information [13]. Since serotonin is so far not implicated in ethanol odor recognition or odor coding the manipulation of serotonin signaling making it an ideal neurotransmitter system to identify novel regulatory mechanism of the attraction to ethanol.

Commonly used genetic tool to map neuronal circuits and uncover regulatory networks underlying behaviors is the use of UAS-transgenes that block neuronal activity in general such as tetanus-toxins or shibire^ts^ under the control of selective Gal4 drivers [14]. However, there is emerging evidence that multiple neuroactive molecules such as neuropeptides and neurotransmitters are expressed in the same neuron and therefore blocking neuronal activity might interfere with more than one neuroactive pathway. For example, the serotonergic dorsal paired medial neurons engulfing the mushroom bodies express the neuropeptide amnesiac and the neurotransmitter GABA [15, 13, 16].

To circumvent the limitations of conventional transgenes we manipulated the key regulator of serotonin signaling–the serotonin transporter (dSert) for several reasons. First, the dSert is highly conserved between mammals and *Drosophila* and takes up serotonin into the presynaptic neuron after release [17, 18]. Second, dSert is exclusively expressed in serotonergic neurons [19], and third, the transformation of non-serotonergic neurons into serotonergic neurons by overexpression of an UAS-dSert transgene is highly unlikely because this transformation depends on excess serotonin levels not present in animals [20]. Finally this tool allows fine-tuning of serotonin signaling by blocking serotonin reuptake, thereby mimicking the effect of serotonin reuptake inhibitors.

To identify serotonergic mechanism regulating the attraction to ethanol, we generated a UAS-dSert transgene with mutated serotonin binding sites resulting in inhibited reuptake and prolonged serotonin signaling in the synaptic cleft. To narrow down serotonergic neurons involved in the regulation of the ethanol attraction we generated a novel serotonergic driver with restricted Gal4 expression in a small subset of serotonergic neurons. Functional studies using an opto-genetics revealed that four neurons suppress the attraction to ethanol. An additional set of two neurons counteracts this suppression. The results uncover further a hierarchy of what kind of information influences the behavioral outcome.

## Material and Methods

### Fly stocks

The following *Drosophila* lines were used: *w*^*1118*^ *norp*A^1^; UAS-*ChR2*; UAS-*ChR2* [21]; *Tph*-Gal4 [22]; *Trh-*Gal4 [23]; *RN2-E-*Gal4 [24]; and UAS-mCD8::GFP [25]. To reduce the influence of genetic background in the behavioral experiments, transgenic fly lines were backcrossed to the *w*^*1118*^ (Scholz lab) for five generations. Flies were raised on standard agar–cornmeal–yeast food at 25°C and 60% relative humidity on a 12 h:12 h light–dark cycle.

### Generation of constructs

To generate *Sert3*-Gal4, the primers 5′-GGT CCA ATC CAA TGG TGT AC-3′ (-909) and 5′-CAT CGT CCT CGT TGG AGT-3′ (+1082) were used to generate fragment F1 containing a *Pvu*II site. A second fragment was generated using 5′-AAT TTC CAC GAC CAC AGG G -3′ (+728) and 3′CAG GGA TTA TCG CAC GAC-3′ (+3010) containing the same *Pvu*II site. Both fragments were cloned into pCRII-TOPO vector. Fragment 1 was cut out using the 5′ flanking *Asp718* site and internal *Pvu*II site, whereas fragment 2 was cut out using the internal *Pvu*II site and flanking *Not*I site. Both fragments were cloned into an *Asp718* and *Not*I—predigested p221-4-Gal4 vector. The *Sert3*-Gal4 insertion line #97 was used for analysis.

To generate a pUAS-*gfp-sert*^*DN*^ construct, the dSert cDNA of the clone RE10485 was sub-cloned via *Asp718* and *Xma*I digest into the pBSII KS (+) vector. Using a T7 primer and the *Xba*I antisense primer 5′-GTC GAG GCA ACC TCT AGA ATA GAA CTG GCC CAG-3′, the first 889 nucleotides were amplified, and the following 2327 nucleotides of cDNA were amplified using the T3 primer and *Xba*I sense primer 5′-CTG GGC CAG TTC TAT TCT AGA GGT TGC CTC AGC-3′. Both fragments were linked via the *Xba*I site, predigested with *Not*I and *Asp718*, and inserted into a *Not*I/*Asp718* predigest pUAS-*gfp-NotI/Asp718* vector.

### Behavioral analysis

Olfactory ethanol preference was assayed as described in [4]. Briefly, a population of 50–80 two- to four-day-old male flies was given the choice between two odor traps placed within a glass beaker at 25°C and 60% relative humidity. Both odor traps were filled with mango–apple juice (Alnatura, Germany GTIN: 4104420071841), and one trap was supplemented with 5% final concentration of ethanol (> 99% EtOH, VWR). The preference index (PI) was calculated by subtracting the number of flies in the odor trap containing ethanol from the number of flies in the ethanol-free odor trap and dividing by the total number of flies. Data presents mean ± s.e.m.

Behavioral changes different from random choice were determined by using a one-sample sign test and were considered as attraction. Lowercase “a” above bars indicates a significant difference from random choice (*P* < 0.05). The behavioral data are summarized in S2 Table.

To activate neurons with blue light, experimental male *norp*^*A1*^; UAS-*ChR2/Sert3-Gal4*; UAS-*ChR2* flies were raised on standard food vials containing 150 μl of ethanol or 150 μl of 250 mM all-*trans*-retinal (R2500; Sigma-Aldrich, Germany) dissolved in ethanol. After hatching, 80 male flies were sedated with CO_2_, collected, and transferred into food vials with ethanol only or with all-*trans*-retinal in ethanol and were kept in the dark. Two days after recovery from CO_2_ sedation at 25°C, three—to five-day-old flies were tested for preference.

The light activation setup is described in [8]. Briefly one odor trap was illuminated with a blue light with a blue LED (465–485 nm; Cree, Germany); a second trap was illuminated with yellow light which was generated by a warm-white LED (Cree, XLAMP, XR_E LED with 2,600 k-3,700K CCT) and a 510-nm blue light filter (HEBO, Aalen, Germany), that takes out all visible wavelengths up to 510-nm. Heat generation by light depends on the light intensity. Therefore we calibrated both diodes to the same intensity of 1800 lx before every test. The following sequence of light pulses was applied by both LEDs: 2 s of 40Hz, 16 s of 8Hz, and 2 s of no light. The number of flies in each trap was determined after all flies had entered a trap. Light preference was calculated as the number of blue light-illuminated flies minus the number of yellow light-illuminated flies divided by the total number of flies.

### Immunohistochemistry

Immunostaining was performed as described previously [8]. The following antibodies were used: rabbit anti-GFP (1:1000, Life Technologies), chicken anti-GFP (1:1000, Life Technologies), rat anti-5HT (1:100, Millipore), mouse anti-nc82 (1:20, DSHB), Alexa Fluor 488 goat anti-chicken IgG (1:1000, Life Technologies), Alexa Fluor 488 goat anti-rabbit IgG (1:1000, Life Technologies), Cy3 goat anti-rat IgG (1:200, Jackson Immunoresearch), and Alexa Fluor 546 goat anti-mouse IgG (1:500, Life Technologies). Confocal stacks with one μm optical sections were generated with a Zeiss 510 confocal microscope. The ImageJ A.1.44 software was used to analyses cell clusters, to generate Z-projections and to determine staining intensities by pixel intensities. Images were processed using Adobe Photoshop CS5/6.

### Statistics

All data were analyzed using StatView 5.0.1 (SAS Institute, Cary, NC, USA) and Statistica 9.1 (StatSoft, Tulsa, OK, USA). A nonparametric one-sample sign test was used to test whether the outcomes were significantly different from random choice. ANOVA with post hoc Tukey–Kramer test was used to test for significant differences among experimental groups. *P < 0.05; **P < 0.01, ***P < 0.001.

## Results

**Fig 1:**
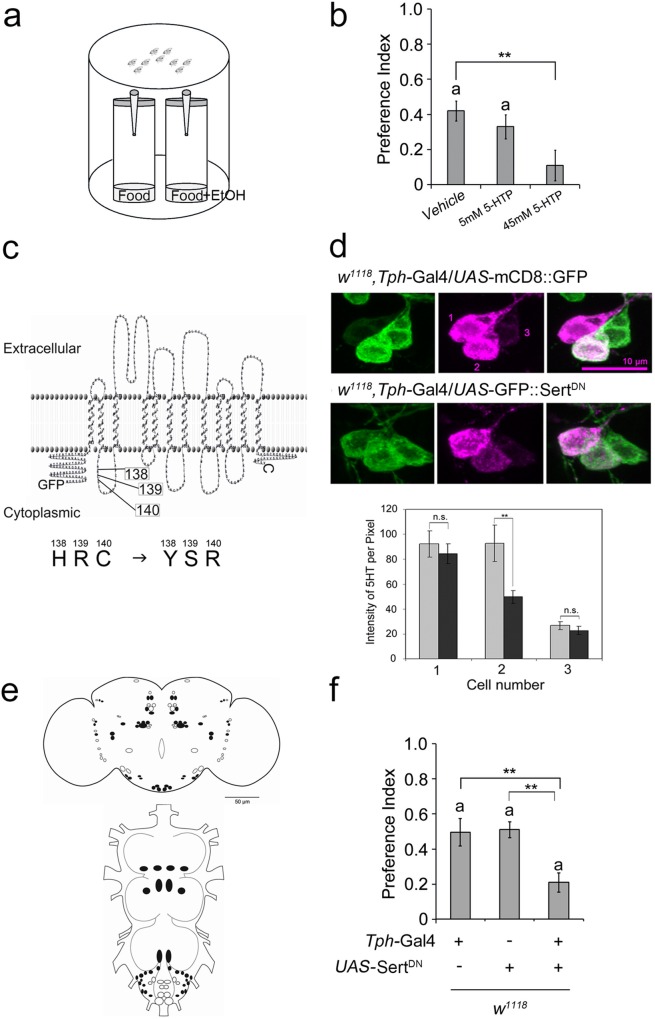
Serotonin regulates olfactory ethanol preference. **a**, Binary choice assay. **b**, Wild-type flies fed 5-HTP showed reduced attraction to ethanol (*n* = 26–30). **c,** Mutated dSert protein encoded by *UAS-Sert*^*DN*^ transgene. **d,** Expression of *UAS*-Sert^DN^ in a *Tph*-Gal4 dependent manner reduces serotonin immunoreactivity in SE3 and T1 of 3rd-instar brain (*n* = 5 larvae). **e,** Serotonin-positive neurons targeted by *Tph*-Gal4 are indicated by filled dots. **f,** Attraction to ethanol is reduced by expression of *UAS*-Sert^DN^ under the control of the *Tph*-Gal4 driver (*n* = 21). *P < 0.05; **P < 0.01. Lowercase **a** above bars indicates attraction (a significant difference from random choice; *P* < 0.05). Bars show mean ± s.e.m.

To test olfactory ethanol attraction in flies, we use a binary choice assay consisting of two odor traps filled with the odor of mango–apple juice [4]; Fig 1A). In addition, one odor trap is enriched with 5% ethanol (EtOH). The attraction of the fly population is determined by the number of flies entering the ethanol-enriched odor trap in comparison to the total number of flies and is reflected by a preference index (PI) that differs from random choice. Control flies normally prefer ethanol-enriched food odors to plain food odors, which are reflected by a PI of ~ 0.4 (Fig 1B).

### Serotonin modulates attraction to ethanol

To address whether serotonin regulates olfactory attraction to ethanol, we increased serotonin levels pharmacologically by feeding the serotonin precursor 5-hydroxytrypthophan (5-HTP) to control flies (Fig 1B), a procedure resulting in a measurable increase in serotonin concentration in fly heads [26]. Increasing 5-HTP concentration resulted in a significant loss of preference. To further explore the function of serotonin in ethanol attraction at the central brain level, we wanted to change serotonin signaling specifically in serotonergic neurons using the UAS/Gal4 system [27]. Therefore, we decided to block serotonin reuptake by manipulating the *Drosophila* serotonin transporter dSert. There is evidence that Serts function as oligomers [28, 29]. Thus over-expressing a mutated version of the transporter under UAS control will interfere with the endogenous dSert levels and function.

To generate a non-functional UAS-*dSert* transgene, we considered that in rats the conserved amino acids (AA) His143 and Asp145 are essential for serotonin binding and serotonin transporter activity [30]. The corresponding AAs in dSert—His138 and the two additional flanking Arg 139 and Cys140—were replaced by tyrosine, serine, and arginine, respectively (Fig 1C). To test whether this mutated dSert protein indeed blocks reuptake into the presynaptic neuron (e.g. effectively reduces serotonin concentrations in the presynaptic serotonergic neuron), we took advantage of Gal4 expression differences in the *Tph*-Gal4 line [22] in the third sub-esophageal and first thoracic segment in the third instar larvae (SE3 and T1 respectively; Fig 1D). Normally, three serotonergic soma per hemisphere can be identified; two neurons show strong expression of 5-HT, and one shows weak expression [19]; Fig 1D. The expression of the UAS-mCD8::GFP transgene [25] driven by the *Tph*-Gal4 line illustrates differences in Gal4 levels in the soma of these serotonergic neurons. Neuron 1, with high serotonin levels, expresses low levels of Gal4/GFP and therefore can only be weakly modified by transgene expression; this neuron serves as an internal control for serotonin levels. Neuron 2 expresses high levels of Gal4/GFP and serotonin. Here serotonin levels can be modified strongly by transgene expression. In contrast, neuron 3 expresses high levels of Gal4/GFP but only modest levels of serotonin. The expression of the mutated UAS-*dSert* transgene resulted in a significant reduction of serotonin staining in neuron 2 in comparison to the internal control neuron 1 (Fig 1D). The expression of the modified dSert efficiently reduced internal presynaptic serotonin levels. This is due to the block of 5-HT up-take from the synapse by the mutated dSert. Therefor 5-HT stays longer in the synapse after release and prolongs 5HT signaling. Since monoamine transporters like Sert functions in a multimeric fashion [31], our mutated version functions as a dominant negative dSert (dSert^DN^). Blocking serotonin uptake by expression of UAS-Sert^DN^ in a *Tph*-Gal4-dependent manner in 54% of the serotonergic neurons significantly reduced olfactory attraction to ethanol (Fig 1E and 1F; S1 Table). Together with the observation that pharmacological elevation of serotonin levels resulted in reduced preference, these results indicate that prolonged serotonin signaling in the synaptic cleft of a subset of serotonergic neurons reduces olfactory attraction to ethanol.

### Subsets of neurons mediates ethanol attraction

**Fig 2:**
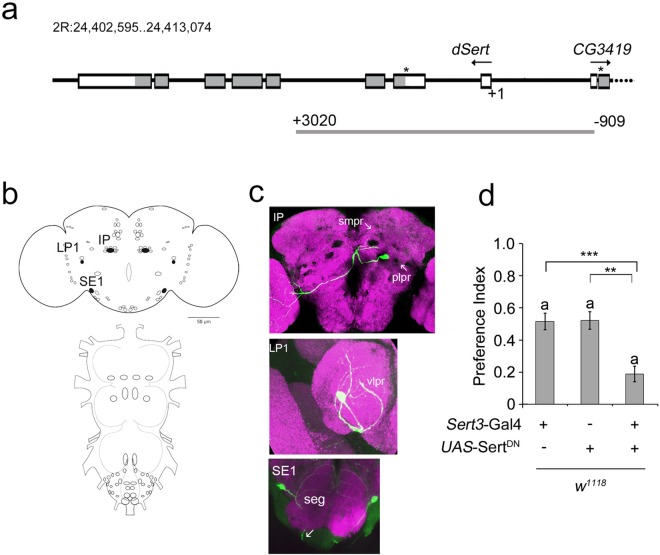
**Reduced ethanol attraction by prolonged serotonin signaling a,** Genomic organization of *dSert*. +1, transcription start site; *, translation start site. Coding exons are in dark grey; arrows indicate direction of transcription relative to annotation. Light grey bar marks genomic fragment used to generate *Sert3*-Gal4. **b**, Serotonergic neurons targeted by the *Sert3*-Gal4 driver are indicated by filled circles. **c,** In an adult brain, nC82 antigen in magenta marks neuropil areas and green transgene expression driven by *Sert3*-Gal4. **d,** Expression of UAS-Sert^DN^ in a *Sert3*-Gal4 dependent manner reduces attraction (*n* = 15–27).

To refine the subset of dSert-expressing neurons mediating attraction to ethanol, we generated the *Sert3*-Gal4 driver containing a 3.9-kb genomic fragment including the *dSert* transcription start site (Fig 2A). Visualizing the Gal4 expression domain of the *Sert3*-Gal4 line using a UAS-mCD8::GFP or UAS-tau::GFP transgene with serotonin immunoreactivity (IR), we identified three serotonergic neurons per head hemisphere that express GFP (Fig 2B and 2C). These consist of one neuron each in the SE1, LP1, and IP clusters (Fig 2B and 2C). The IP neuron projects to the smpl, plpr, and optic system; the SE1 neuron innervates the subesophageal ganglion (seg) and projects into the head and the LP1 neuron is vlpr intrinsic. Expression of the impaired Sert function using the UAS-Sert^DN^ transgene in these neurons impaired olfactory attraction to ethanol (Fig 2D). Comparing the expression patterns of the *Tph*-Gal4 and *Sert3*-Gal4 drivers, common co-expression of GFP and serotonin is found in the cells of the IP, SE1, and LP1 clusters (compare S1 Table; Fig 2B and S1B Fig). Different SE1 neurons are targeted by *Tph*-Gal4 and *Sert3*-Gal4; therefore, the comparison supports that IP and/or LP1 neurons regulate this attraction.

**Fig 3:**
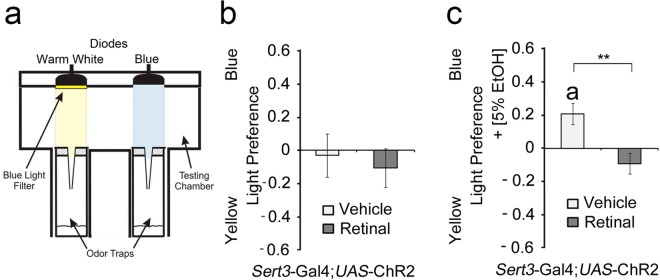
Behavioral inhibition by serotonin. **a,** Odor trap assay paired with an opto-genetic set up. **b,** No altered preference after blue-light activation of *Sert3*-Gal4 and UAS-ChR2 neurons was observed (*n* = 22–26). **c**, Activation of *Sert3*-Gal4-dependent neurons reduces attraction (*n* = 30). The errors are s.e.m.

Innate olfactory attraction to ethanol depends on the action of a positive and/or negative reinforcer [8]. To address whether elevation of serotonin in the synaptic cleft of LP1 and IP neurons is sufficient to induce attraction, we activated these neurons using a UAS-channel rhodopsin transgene (*ChR2*) in a *Sert3*-Gal4 dependent manner (Fig 3). Channel rhodopsin is a light-sensitive cation channel that can be activated by blue light in the presence of *trans*-retinal, resulting in neuronal activation when expressed in neurons [32]. We used the same frequency of blue light stimulation that resulted in site preference when octopaminergic/tyraminergic neurons were activated [8]. Flies were given a choice between two identical odor sources (Fig 3A and 3B), one of which was illuminated with blue and one with yellow light with the same intensity to account for differences in heat generation. When flies enter the region of blue light illumination, neurons expressing ChR2 are activated; yellow light does not cause activation. Here, the activation of serotonergic neurons did not result in site preference. Since under the same experimental condition the activation of octopaminergic neurons resulted in attraction and activation of dopaminergic neurons in aversion (Schneider et al., 2012), the low penetrance of blue light through the cuticle do not account for the loss of site preference. Other factors that might influence attraction include mechanisms that inhibit the execution of the approach and therefore allow the animal to dynamically adjust its goal-oriented behaviors [99. Serotonin has been implicated in the neuronal basis of response control in humans and other mammals [9, 33, 34]. To address whether serotonin indeed inhibits the execution of approach, we next activated serotonergic neurons in the presence of one food odor trap with and one without 5% EtOH (Fig 3C). As expected, control flies preferred food odors containing ethanol. In contrast, activation of serotonergic neurons abolished preference.

Therefore, these neurons are sufficient to negatively regulate attraction to ethanol, and developmental alteration of long-term expression of the mutated dSert cannot account for this inhibition.

### Counteracting neurons regulate attraction

**Fig 4:**
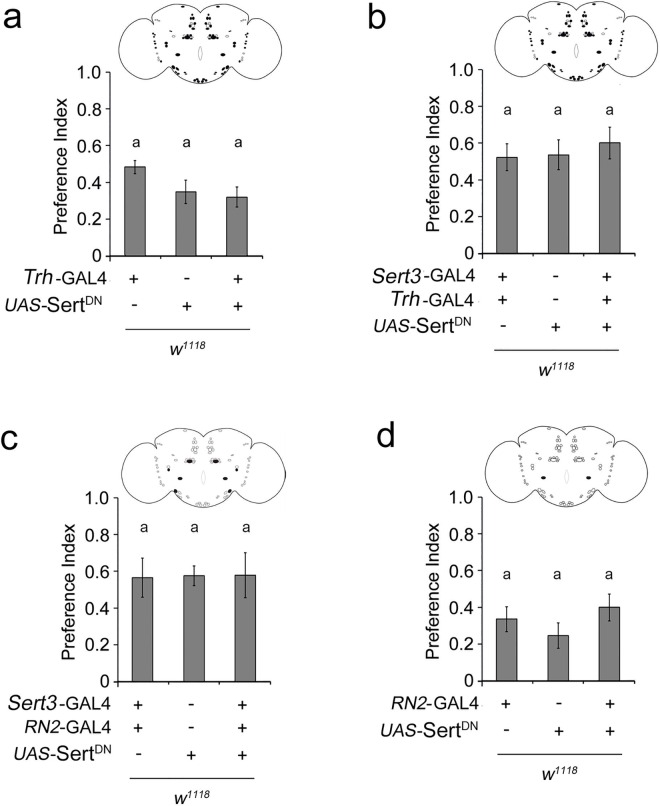
**Regulation of attraction by two counteracting serotonergic mechanisms a**, Attraction to ethanol is not altered by expression of *UAS*-Sert^DN^ under control of *Trh*-Gal4 (*n* = 30–31); or **b**, *Trh*-Gal4; *Sert3*-Gal4 drivers (*n* = 31–32); or **c**, *Sert3*-Gal4; *RN2*-Gal4 drivers (*n* = 30–31). **d**, Expression of Sert^DN^ in CSD neuron does not alter preference (*n* = 45–58). **a–d**, Black dots in schematic summarize targeted serotonergic neurons. *P < 0.05; **P < 0.01. Bars show mean ± s.e.m.

To identify additional serotonergic neurons regulating the attraction to ethanol, we expanded the expression of dSert^DN^ to a broader set of neurons using the *Trh*-Gal4 line ([23]; Fig 4A). This driver targets transgene expression to approximately 83% of serotonergic neurons ([23]; S1 Table and S2 Fig). Expression of dSert^DN^ in this set of serotonergic neurons did not alter attraction (Fig 4A). To rule out the possibility that the strength of the driver might not be sufficient to express high levels of mutated Sert, we combined the *Trh*-Gal4 line with the *Sert3*-Gal4 driver (Fig 4B and S3 Fig). Under the control of both Gal4 drivers, the expression of *UAS-*Sert^DN^ again did not interfere with olfactory ethanol preference, supporting the idea of a compensatory serotonergic mechanism that acts against the inhibitory effect of the *Sert3*-Gal4-targeted neurons. One way in which ethanol preference could be modulated is by strengthening of the sensory input that elicits the behavior. In *Drosophila*, serotonin enhances calcium signaling in response to odors in the glomeruli of the antennal lobes, the first center where olfactory information might be altered by additional modifying neurons [12]. The antennal lobes are innervated by two contralateral projection serotonin-immunoreactive deutocerebral (CSD) neurons, which are the only serotonergic neurons innervating this structure and which regulate olfactory sensitivity in an odorant-dependent manner [35,36, 37]. The expression domain of *Trh*-Gal4 includes these neurons, but those of *Sert3*-Gal4 and *Tph*-Gal4 do not (S1 Table), making the CSD neurons excellent candidates for this compensation. The CSD neurons can be exclusively targeted by the *RN2*-Gal4 driver ([24]; Fig 4C and S5 Fig). To address whether prolonged serotonin signaling in the CSD neurons reverses the reduced olfactory attraction caused by changed serotonin signaling in the LP1 and/or IP neurons, we extended the Gal4 expression of *Sert3*-Gal4 to the CSD neurons by combining the *RN2*-Gal4 driver with *Sert3*-Gal4. Expression of Sert^DN^ in this extended neuron set did not alter normal levels of olfactory attraction to ethanol (Fig 4C and S4 Fig). Therefore, reduced serotonin levels in the CSD neuron compensate for the inhibitory effect of the LP1 and/or IP neurons.

**Fig 5:**
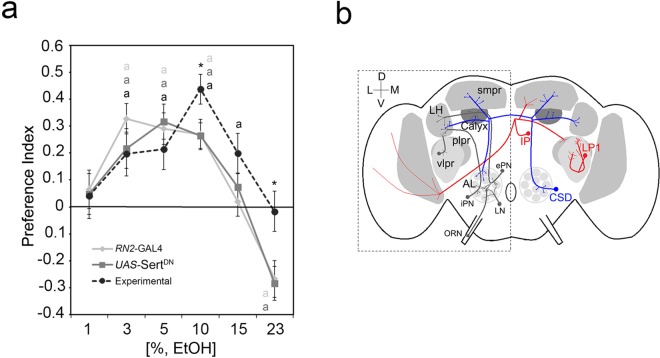
**Regulation of attraction by two counteracting serotonergic mechanisms a**, Expression of Sert^DN^ in CSD neuron increases preference for 10% ethanol-enriched food odors. Lowercase **a** indicates preference (*P* < 0.05). The errors present s.e.m. **b**, Model of **serotonergic** neurons modulating olfactory ethanol preference.

This compensation could be due to enhanced olfactory attraction. To test this, we analyzed whether flies with altered serotonin signaling in the CSD neurons have enhanced preference for food odors containing 5% EtOH (Fig 4D), and no enhanced preference was observed. Alternatively, the information processed by the CSD neurons might bypass the inhibitory function of the IP and/or LP1 neurons by enhancing the odor information from the antennal lobes to the mushroom bodies, the lateral horn and the contralateral antennal lobe changing the stability of the odor information. It has been suggested that serotonin influences the coding stability of the odor at the level of the antennal lobe glomeruli in *Drosophila* [12] and that the innate olfactory attraction is already encoded at this level [38]. To test whether the stability of positive olfactory information might be shifted, we analyzed olfactory attraction to ethanol in flies with prolonged serotonin signaling in the CSD neurons at different EtOH concentrations (Fig 5A). Flies did not show greater olfactory attraction at low EtOH concentrations, ruling out a shift in sensitivity (Fig 5A). In contrast, we found that prolonged serotonin signaling in CSD neurons resulted in increased attraction to food odors containing 10% EtOH. This observed shift in attraction to higher EtOH concentration could be due to increased resistance in olfactory receptor neurons. If this is true, also a delay in response to low EtOH should be observed. However, the threshold for recognizing lower ethanol concentrations is not increased and the degree of attraction towards food odors containing 5% EtOH is also not changed by altered serotonin signaling (Figs 4C and 5A). Thus, the results are consistent with changes in the coding stability of positive odor information by prolonged serotonin signaling of the CSD neuron.

## Discussion

Olfactory attraction to ethanol is regulated by two sets of serotonergic neurons. Two neurons—the serotonergic CSD neurons—counteract the inhibitory function of a second set of four serotonergic neurons. The enhancement of sensory input by altered serotonin signaling of the CSD neurons is stronger than more internal information that might influence the behavioral outcome. The observation that strong sensory information overrules internal conditions unravels a hierarchy of information that governs behavioral output.

Prolonged serotonin signaling in approximately 83%of the serotonergic neurons using the *Trh*-Gal4 driver results in normal degree of attraction to 5% EtOH containing food odors. At the first glance, these finding suggest that dSert in these broad set of serotonergic neurons is not involved in the regulation of ethanol attraction. This apparently normal behavior is shared by other *Sert* knock out animals. For example *Sert* knock out mice develop normal conditioned place preference–a model for the cocaine reward—despite the fact that cocaine binds to Sert [39, 40]. However, breaking down the serotonergic networks underlying the regulation of the attraction to ethanol revealed that a dysregulation of serotonergic neurons can phenocopy normal behavior and that the identified serotonergic neurons under normal circumstance indeed regulate the olfactory attraction. The results show that compensation can occur on network level. The serotonergic neurons underlying the gating of olfactory attraction to ethanol innervate distinct brain regions normally involved in the regulation of olfactory responses (Fig 5B). The attraction promoting serotonergic CSD neuron receives synaptic input from the ipsilateral antennal lobes from a broad set of glomeruli, collecting and exchanging olfactory information with the mushroom bodies and lateral horn and releasing information to the contralateral antennal lobe regions [36].

The second set of inhibitory serotonergic neurons includes one neuron each from the IP and LP1 clusters that suppresses innate attraction to ethanol odor. The neuron of the IP cluster projects into the superior medial protocerebrum (smpr), posterior lateral protocerebrum (plpr), and the optic system. The smpr receives olfactory output both directly from the antenna lobes and indirectly from lobes of the LH [41]. The LP1 neuron innervates the ventral lateral protocerebrum (vlpr), a region implicated as a multimodal integration center of sensory stimuli including olfactory information [42, 43 and 44]. This multimodal nature of this brain region is further supported by recent finding that a pair of similar serotonergic neurons is involved in aggression [23] and mediating the hunger state of the fly [45]. The fact that the alteration of serotonin signaling in the CSD neuron bypasses the inhibitory effect of the *Sert3*-Gal4-dependent neurons further supports that the inhibitory effect of those neurons is not due to loss of olfactory perception. Combined with the observation that activation of the *Sert3*-Gal4-dependent neurons alone does not cause attraction or aversion, these results show that these neurons regulate the execution of a function rather than being involved in the initial step of odor information processing or execution of the approach behavior. This is consistent with a role for prolonged serotonin signaling in response inhibition [9]. This suppression of innate olfactory attraction to ethanol can be overruled by strengthening the constant sensory input to the olfactory system. The consequence of the failing inhibitory mechanism is that the likelihood of exposure to food odors containing ethanol increases, and the opportunity to ingest ethanol might increase in turn.

The observation that strong sensory information overrules internal conditions also suggests that under normal circumstances both mechanisms require a tight temporal control. That timing matters is further substantiated by the observations that short term systemic application of a serotonin precursor and short term activation of the Sert3-Gal4 dependent subset of serotonergic neurons using opto-genetics reduce ethanol attraction (Figs 1B and 3C), but long term intervention of serotonin signaling by dSert^DN^ expression under the control of the Trh-Gal4 driver did not interfere with ethanol attraction (Fig 4A). However the regulation of innate ethanol odor attraction is even more complex since other neurotransmitter systems are also involved in regulating the approach to an olfactory stimulus. We have previously shown that olfactory ethanol attraction depends on the reinforcement of the olfactory information by a subset of octopaminergic/tyraminergic neurons and the aversion depends on the activation of a subset of dopaminergic neurons acting as a negative reinforcer [8]. Here, we show that the maintenance of olfactory information by the serotonergic CSD neurons promote the approach to the ethanol food odor mixture. In the presence of prolonged external olfactory information, the inhibitory information of an internal serotonergic reference system is overruled. The regulation of behavioral outcome requires a tight temporal regulation between the positive and negative reinforcing, inhibiting and prolonging mechanisms depending on the location of the fly relative to the ethanol containing food source and its internal condition. It will be interesting to see where the four mechanisms intersect with each other, how they are orchestrated and whether they are sufficient to regulate the attraction to ethanol. Furthermore, given the evolutionarily conserved nature of the serotonin transporter and the wide expression of serotonin, the serotonergic gating mechanism might delineate a basic principle for other behaviors and organisms as well.

## Supporting Information

S1 Table Comparative neuroanatomical analysis of Gal4 expression domain of different serotonergic Gal4 drivers. For nomenclature see [19]. Numbers reflect counted soma; *n* is number of analysed clusters.
(PDF)

S2 Table Original data related to Figs 1, 2, 3, 4 and 5. (PDF)

S1 Fig Comparative serotonin IR and GFP expression of Tph-Gal4 driver. **a–d**, Magenta shows serotonin IR, and green shows GFP expression of UAS-md8::GFP transgene. Images are merged in **a, a**′ and **b**′′-**d**″. **a**, Z-projection of the anterior and **a′**, posterior part of an adult male brain shows co-expression of serotonin and GFP in white. **b–b″**, Higher magnification shows two neurons in the SE1 cluster of the adult brain that express GFP and serotonin. **c**, Serotonergic neurons expressing Gal4 are found in the pro-, meta- and mesothoracic segments. **d**, In the abdominal segment (Abdm), an average of 11 cells express GFP and serotonin. Neurons expressing serotonin or GFP only are marked with an arrow or star, respectively. See [19] for nomenclature.
(PDF)

S2 Fig Comparative serotonin IR and GFP expression of *Trh*-Gal4 driver. **a–d**, Serotonin IR is labelled in magenta, GFP is labelled in green, and co-expression of serotonin and GFP is shown in white. Images are merged in **a, a′** and **b″d″**. **a**, Z-projection of the anterior and **a′**, posterior part of an adult male brain. **b–b″**, Serotonergic neurons expressing Gal4 in the pro-, meta- and mesothoracic segments. **c–c″**, A more ventral view and **d–d″**, dorsal view of the abdomen (Abdm). Neurons expressing serotonin or GFP only are marked with a small open circle or star, respectively. See [19] for nomenclature. The schematic summarizes the data presented in S1 Table; black circles indicate serotonergic neurons that express GFP, and empty circles indicate those that express serotonin only.
(PDF)

S3 Fig *Trh;Sert3*-Gal4 drivers express GFP transgenes in almost all serotonin IR-positive neurons. **a–d**, Serotonin IR is labelled in magenta, GFP is labelled in green, and co-expression of serotonin and GFP is shown in white. Images are merged in **a**, **a′**and **b″– e″**. **a**, Z-projection of the anterior and **a′**, posterior part of an adult male brain. **b–b″**, Serotonergic neurons expressing Gal4 in the pro-, meta- and mesothoracic segments. **c–e,** The Z-stacks of the abdomen (Abdm) are divided into three equal parts for better resolution. **c–c″**, A more ventral view; **d–d″**, a more medial view; and **e–e″**, a more dorsal view. See [19] for cluster nomenclature. The schematic summarizes the data presented in S1 Table; black circles indicate serotonergic neurons that express GFP, and empty circles indicate those that express serotonin only.
(PDF)

S4 Fig Serotonin IR and GFP expression of *Sert3*, *RN2*-Gal4 drivers. **a–c**, Serotonin IR is labelled in magenta, GFP is labelled in green, and co-expression of serotonin and GFP is shown in white. Images are merged in **a**, **a′**, **b″to c″**. **a,** Z-projection of the anterior and **a′**, posterior part of an adult male brain. **b–b″**, Pro-, meta- and mesothoracic segments with serotonergic neurons expressing Gal4. **c–c″**, A more detailed view of the abdomen (Abdm). The schematic summarizes the data presented in S1 Table; black circles indicate serotonergic neurons that express GFP, and empty circles indicate those that express serotonin only.
(PDF)

S5 Fig The *RN2*-Gal4 driver expresses GFP only a sparse set of serotonergic neurons. **a–c**, Serotonin IR is labelled in magenta, GFP is labelled in green, and co-expression of serotonin and GFP is shown in white. A merge is shown in **a**, **a′**, **b″to c″**. Z-projection of the **a**, anterior and **a′**, posterior part of an adult male brain. **b–b″**, Pro-, meta- and mesothoracic segments with serotonergic neurons expressing Gal4. A more detailed view of the abdomen (Abdm). The schematic summarizes the clusters expressing serotonin and GFP in black circles and serotonin only expressing cells with empty circles.
(PDF)
