# Correction: A Single Pair of Serotonergic Neurons Counteracts Serotonergic Inhibition of Ethanol Attraction in *Drosophila*

**DOI:** 10.1371/journal.pone.0174010

**Published:** 2017-03-09

**Authors:** Li Xu, Jianzheng He, Andrea Kaiser, Nikolaus Gräber, Laura Schläger, Yvonne Ritze, Henrike Scholz

The fourth author’s name is spelled incorrectly. The correct name is: Nikolaus Gräber.
