# Supplementary material for: A Single Pair of Serotonergic Neurons Counteracts Serotonergic Inhibition of Ethanol Attraction in Drosophila
Source: PLoS One. 2016 Dec 9;11(12):e0167518. doi: 10.1371/journal.pone.0167518 (PMC5147910; doi:10.1371/journal.pone.0167518)
Supplement: S1 Table — For nomenclature see [19]. Numbers reflect counted soma; n is number of analysed clusters. (PDF) [file pone.0167518.s001.pdf]

***Tph*-Gal4**

Head	**5-HT ± STDEV**	**GFP ± STDEV**	**Merge ± STDEV**	n
SP1	3.0 ± 0.0	3.7 ± 0.5	2.1 ± 0.3	21
SP2	5.2 ± 0.7	4.9 ± 1.0	2.5 ± 0.7	19
IP	7.5 ± 0.8	8.1 ± 1.3	4.6 ± 0.8	20
LP1	2.0 ± 0.0	5.4 ± 0.8	2.0 ± 0.0	14
LP2	9.8 ± 1.1	12.9 ± 1.9	2.8 ± 0.8	16
LP3	2.8 ± 0.7	16.2 ± 1.7	1.6 ± 1.0	9
SE1	4.1 ± 0.3	4.8 ± 0.9	2.2 ± 0.7	14
SE2	2.1 ± 0.3	2.1 ± 0.3	2.0 ± 0.4	13
SE3	3.0 ± 0.0	7.2 ± 0.9	3.0 ± 0.0	17
AP	2.9 ± 0.3	6.0 ± 2.4	1.0 ± 0.0	11
DP	1.0 ± 0.0	0.0	0.0	16
CSD	1.0 ± 0.0	0.0	0.0	17
Thorax				
Pro	2.2 ± 0.4	2.9 ± 0.3	2.2 ± 0.4	14
Meso	2.1 ± 0.3	2.9 ± 0.3	2.1 ± 0.3	16
Meta	1.1 ± 0.3	2.6 ± 0.5	1.1 ± 0.3	16
Abdomen				
Abdm	16.5 ± 1.2	13.4 ± 2.3	11.0 ± 2.4	14

***Sert3*-Gal4**

Head	**5-HT ± STDEV**	**GFP ± STDEV**	**Merge ± STDEV**	n
SP1	3.0 ± 0.2	0.0	0.0	23
SP2	5.2 ± 0.5	0.0	0.0	19
IP	7.5 ± 0.7	1.3 ± 0.5	1.3 ± 0.5	19
LP1	2.0 ± 0.2	1.3 ± 0.5	1.3 ± 0.5	19
LP2	9.8 ± 1.4	0.0	0.0	25
LP3	2.2 ± 0.4	0.0	0.0	6
SE1	4.0 ± 0.4	1.4 ± 0.5	1.2 ± 0.5	14
SE2	2.2 ± 0.4	0.0	0.0	22
SE3	3.0 ± 0.2	0.0	0.0	30
AP	3.0 ± 0.2	0.0	0.0	24
DP	1.0 ± 0.0	0.0	0.0	27
CSD	1.0 ± 0.0	0.0	0.0	34
Thorax				
Pro	2.2 ± 0.0	0.0	0.0	12
Meso	1.9 ± 0.3	0.0	0.0	10
Meta	1.2 ± 0.4	0.0	0.0	16
Abdomen				
Abdm	16.5 ± 1.6	0.0	0.0	14

***Trh*-Gal4**

Head	**5-HT ± STDEV**	**GFP ± STDEV**	**Merge ± STDEV**	n
SP1	3.0 ± 0.0	3.5 ± 0.7	3.0 ± 0.2	24
SP2	5.2 ± 0.6	4.7 ± 0.9	3.6 ± 0.6	22
IP	6.9 ± 0.7	7.1 ± 1.0	5.2 ± 1.0	19
LP1	2.0 ± 0.0	9.6 ± 1.7	2.0 ± 0.0	22
LP2	9.5 ± 2.5	12.2 ± 2.0	6.7 ± 2.0	27
LP3	2.7 ± 0.6	1.0 ± 1.2	0.3 ± 0.5	16
SE1	4.1 ± 0.3	4.1 ± 0.4	4.1 ± 0.3	28
SE2	2.0 ± 0.2	2.0 ± 0.5	1.9 ± 0.5	22
SE3	3.0 ± 0.0	3.0 ± 0.0	3.0 ± 0.0	23
AP	2.9 ± 0.3	3.7 ± 1.3	2.1 ± 0.5	26
DP	1.0 ± 0.0	1.0 ± 0.0	1.0 ± 0.0	15
CSD	1.0 ± 0.0	1.0 ± 0.0	1.0 ± 0.0	21
Thorax				
Pro	2.2 ± 0.4	2.3 ± 0.5	2.0 ± 0.0	12
Meso	2.0 ± 0.0	3.0 ± 0.0	2.0 ± 0.0	9
Meta	1.1 ± 0.4	2.2 ± 0.4	1.1 ± 0.4	14
Abdomen				
Abdm	16.5 ± 1.6	17.9 ± 1.0	16.1 ± 1.0	13
