# Supplementary material for: A Single Pair of Serotonergic Neurons Counteracts Serotonergic Inhibition of Ethanol Attraction in Drosophila
Source: PLoS One. 2016 Dec 9;11(12):e0167518. doi: 10.1371/journal.pone.0167518 (PMC5147910; doi:10.1371/journal.pone.0167518)
Supplement: S2 Table — (PDF) [file pone.0167518.s002.pdf]

Table S2: Original data related to Fig. 1, Fig. 2, Fig. 3, Fig. 4 and Fig. 5

Fig. 1b				
**Genotype**	**Mean**	**SEM**	**N**	**Condition**
w^{1118}	0.42	0.06	26	Vehicle
w^{1118}	0.33	0.07	28	5mM 5HTP
w^{1118}	0.14	0.08	30	45mM 5HTP

Fig. 1d			
**Genotype**	**Mean**	**SEM**	**N**
w^{1118}; *Tph*-GAL4	0.49	0.08	33
w^{1118}; *UAS*-Sert^{DN}-GFP	0.51	0.05	28
w^{1118}; *Tph*-GAL4, *UAS*-Sert^{DN}	0.21	0.05	35

Fig. 1f			
**Genotype**	**Mean**	**SEM**	**N**
w^{1118}; *Tph*-GAL4	0.49	0.05	28
w^{1118}; *UAS*-Sert^{DN}	0.50	0.04	35
w^{1118}; *Tph*-GAL4, *UAS*-Sert^{DN}	0.20	0.05	32

Fig. 2d			
**Genotype**	**Mean**	**SEM**	**N**
w^{1118}; *Sert3*-GAL4	0.52	0.06	27
w^{1118}; *UAS*-Sert^{DN}	0.53	0.05	15
w^{1118}; *Sert3*-GAL4, *UAS*-Sert^{DN}	0.22	0.06	21

Fig. 3b				
**Genotype**	**Mean**	**SEM**	**N**	**Condition**
NorpA1; Sert3-GAL4; *UAS*-ChR2	-0.02	0.12	25	Vehicle
NorpA1; Sert3-GAL4; *UAS*-ChR2	-0.12	0.10	26	Retinal

Fig. 3c				
**Genotype**	**Mean**	**SEM**	**N**	**Condition**
NorpA1; Sert3-GAL4; *UAS*-ChR2	0.21	0,06	30	Vehicle
NorpA1; Sert3-GAL4; *UAS*-ChR2	-0.09	0,06	30	Retinal

Fig. 4a			
**Genotype**	**Mean**	**SEM**	**N**
w^{1118}; ;*Trh*-GAL4	0.49	0.04	30

w^{1118}; *UAS*-Sert^{DN}	0.35	0.06	31
w^{1118}; *UAS*-Sert^{DN}; *Trh*-GAL4	0.33	0.05	31

Fig. 4b

Genotype	Mean	SEM	N
w^{1118}; *Sert3*-GAL4; *Trh*-GAL4	0.52	0.07	32
w^{1118}; *UAS*-Sert^{DN}	0.54	0.08	32
w^{1118}; *Sert3*-GAL4, *UAS*-Sert^{DN}; *Trh*-GAL4	0.60	0.09	31

Fig. 4c

Genotype	Mean	SEM	N
w^{1118}; *Sert3*-GAL4; *RN2*-GAL4	0.57	0.11	29
w^{1118}; *UAS*-Sert^{DN}	0.58	0.05	33
w^{1118}; *Sert3*-GAL4, *UAS*-Sert^{DN}; *RN2*-GAL4	0.58	0.12	20

Fig. 4d

Genotype	Mean	SEM	N
w^{1118}; ;*RN2*-GAL4	0.33	0.07	45
w^{1118}; *UAS*-Sert^{DN}	0.24	0.07	58
w^{1118}; *UAS*-Sert^{DN}; *RN2*-GAL4	0.40	0.07	50

Fig. 5a

Genotype	Mean	SEM	N	Conditions
w^{1118}; ;*RN2*-GAL4	0.06	0.07	28	1% EtOH
w^{1118}; *UAS*-Sert^{DN}	0.05	0.08	27	
w^{1118}; *UAS*-Sert^{DN}; *RN2*-GAL4	0.04	0.08	26	
w^{1118}; ;*RN2*-GAL4	0.33	0.07	25	3% EtOH
w^{1118}; *UAS*-Sert^{DN}	0.22	0.08	25	
w^{1118}; *UAS*-Sert^{DN}; *RN2*-GAL4	0.20	0.08	20	
w^{1118}; ;*RN2*-GAL4	0.29	0.06	32	5% EtOH
w^{1118}; *UAS*-Sert^{DN}	0.32	0.07	26	
w^{1118}; *UAS*-Sert^{DN}; *RN2*-GAL4	0.22	0.08	27	
w^{1118}; ;*RN2*-GAL4	0.27	0.06	34	10% EtOH
w^{1118}; *UAS*-Sert^{DN}	0.26	0.05	33	
w^{1118}; *UAS*-Sert^{DN}; *RN2*-GAL4	0.44	0.06	34	

w^{1118}; ;*RN2*-GAL4	0.02	0.05	31	15% EtOH
w^{1118}; *UAS*-Sert^{DN}	0.06	0.05	32	
w^{1118}; *UAS*-Sert^{DN}; *RN2*-GAL4	0.21	0.05	33	
w^{1118}; ;*RN2*-GAL4	-0.27	0.07	25	23% EtOH
w^{1118}; *UAS*-Sert^{DN}	-0.28	0.06	23	
w^{1118}; *UAS*-Sert^{DN}; *RN2*-GAL4	-0.02	0.07	22	
