# Supplementary figures and images for: A Single Pair of Serotonergic Neurons Counteracts Serotonergic Inhibition of Ethanol Attraction in Drosophila
Source: PLoS One. 2016 Dec 9;11(12):e0167518. doi: 10.1371/journal.pone.0167518 (PMC5147910; doi:10.1371/journal.pone.0167518)

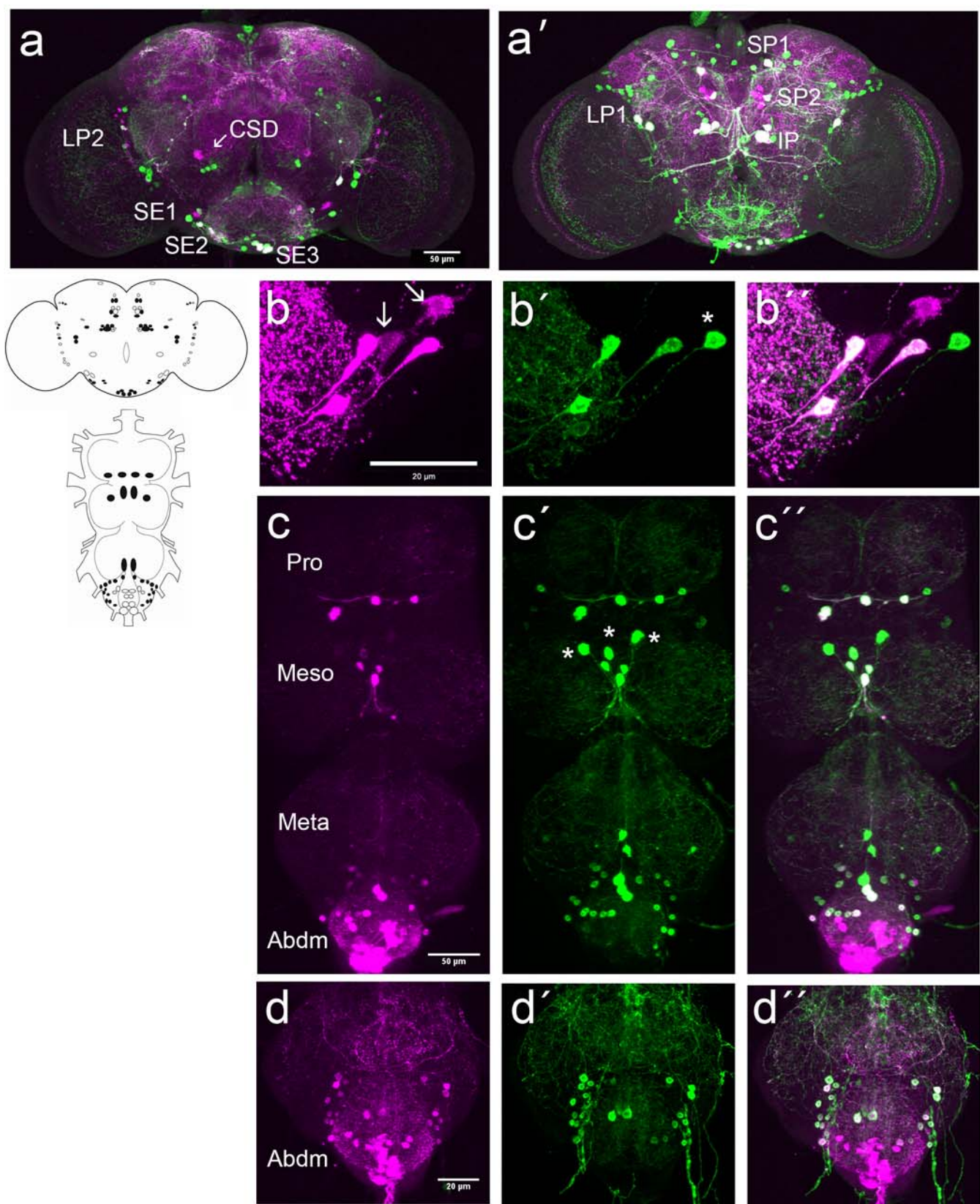

Figure-S1-Scholz

Supplement: S1 Fig — a–d, Magenta shows serotonin IR, and green shows GFP expression of UAS-md8::GFP transgene. Images are merged in a, a′ and b′′-d″. a, Z-projection of the anterior and a′, posterior part of an adult male brain shows co-expression of serotonin and GFP in white. b–b″, Higher magnification shows two neurons in the SE1 cluster of the adult brain that express GFP and serotonin. c, Serotonergic neurons expressing Gal4 are found in the pro-, meta- and mesothoracic segments. d, In the abdominal segment (Abdm), an average of 11 cells express GFP and serotonin. Neurons expressing serotonin or GFP only are marked with an arrow or star, respectively. See [19] for nomenclature. (PDF) [file pone.0167518.s003.pdf]

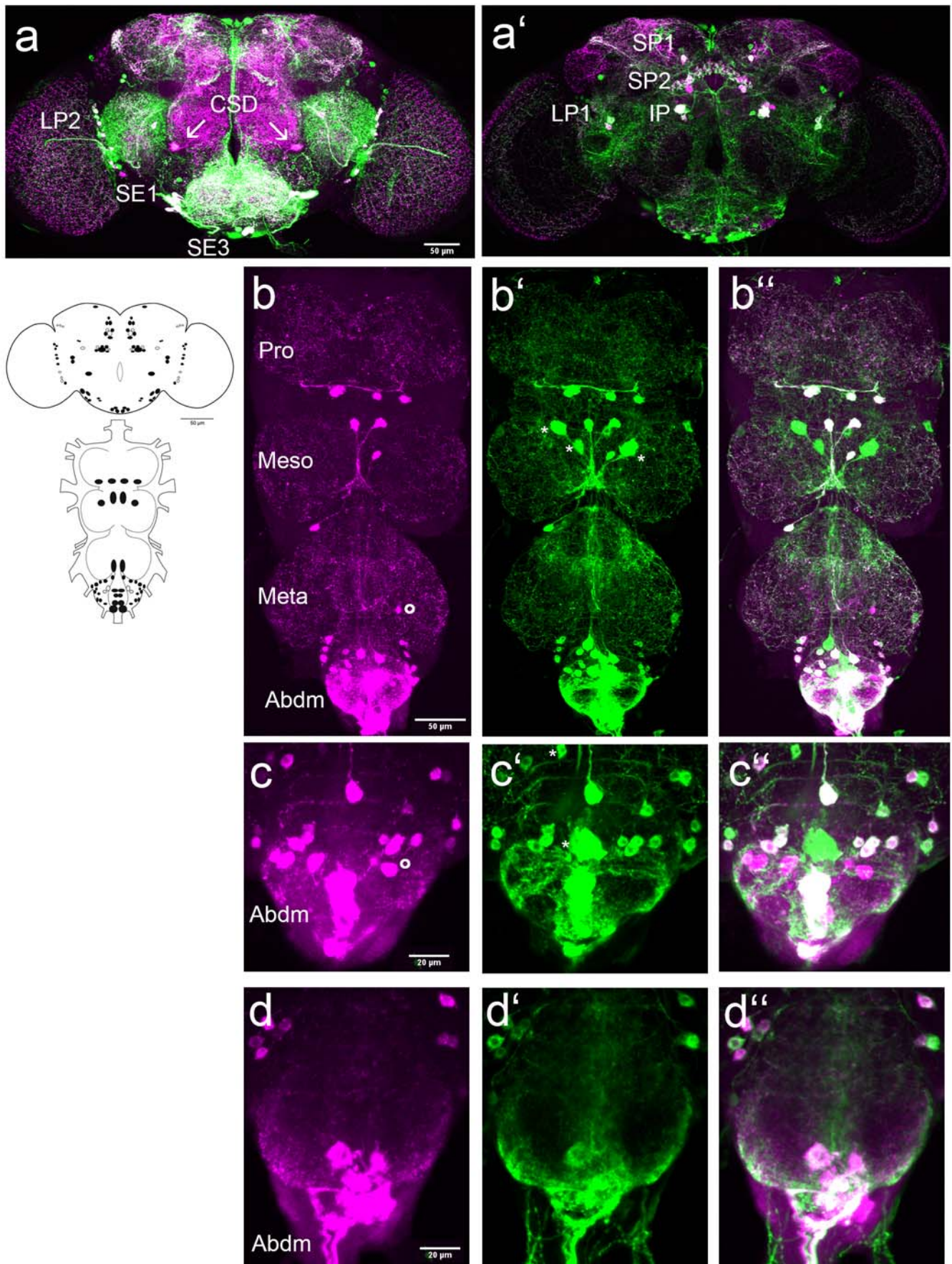

Figure-S2-Scholz

Supplement: S2 Fig — a–d, Serotonin IR is labelled in magenta, GFP is labelled in green, and co-expression of serotonin and GFP is shown in white. Images are merged in a, a′ and b″d″. a, Z-projection of the anterior and a′, posterior part of an adult male brain. b–b″, Serotonergic neurons expressing Gal4 in the pro-, meta- and mesothoracic segments. c–c″, A more ventral view and d–d″, dorsal view of the abdomen (Abdm). Neurons expressing serotonin or GFP only are marked with a small open circle or star, respectively. See [19] for nomenclature. The schematic summarizes the data presented in S1 Table; black circles indicate serotonergic neurons that express GFP, and empty circles indicate those that express serotonin only. (PDF) [file pone.0167518.s004.pdf]

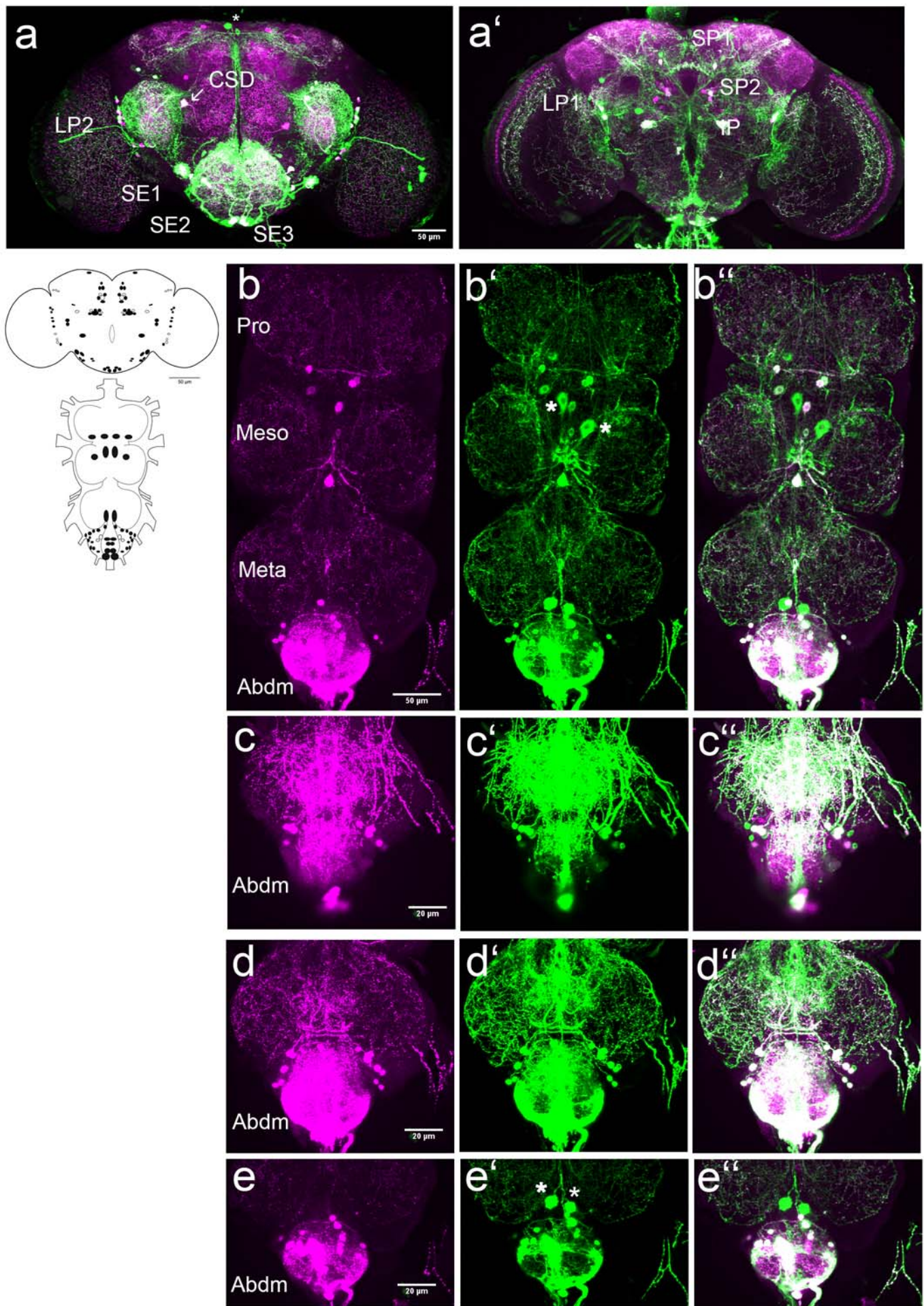

Supplement: S3 Fig — a–d, Serotonin IR is labelled in magenta, GFP is labelled in green, and co-expression of serotonin and GFP is shown in white. Images are merged in a, a′and b″– e″. a, Z-projection of the anterior and a′, posterior part of an adult male brain. b–b″, Serotonergic neurons expressing Gal4 in the pro-, meta- and mesothoracic segments. c–e, The Z-stacks of the abdomen (Abdm) are divided into three equal parts for better resolution. c–c″, A more ventral view; d–d″, a more medial view; and e–e″, a more dorsal view. See [19] for cluster nomenclature. The schematic summarizes the data presented in S1 Table; black circles indicate serotonergic neurons that express GFP, and empty circles indicate those that express serotonin only. (PDF) [file pone.0167518.s005.pdf]

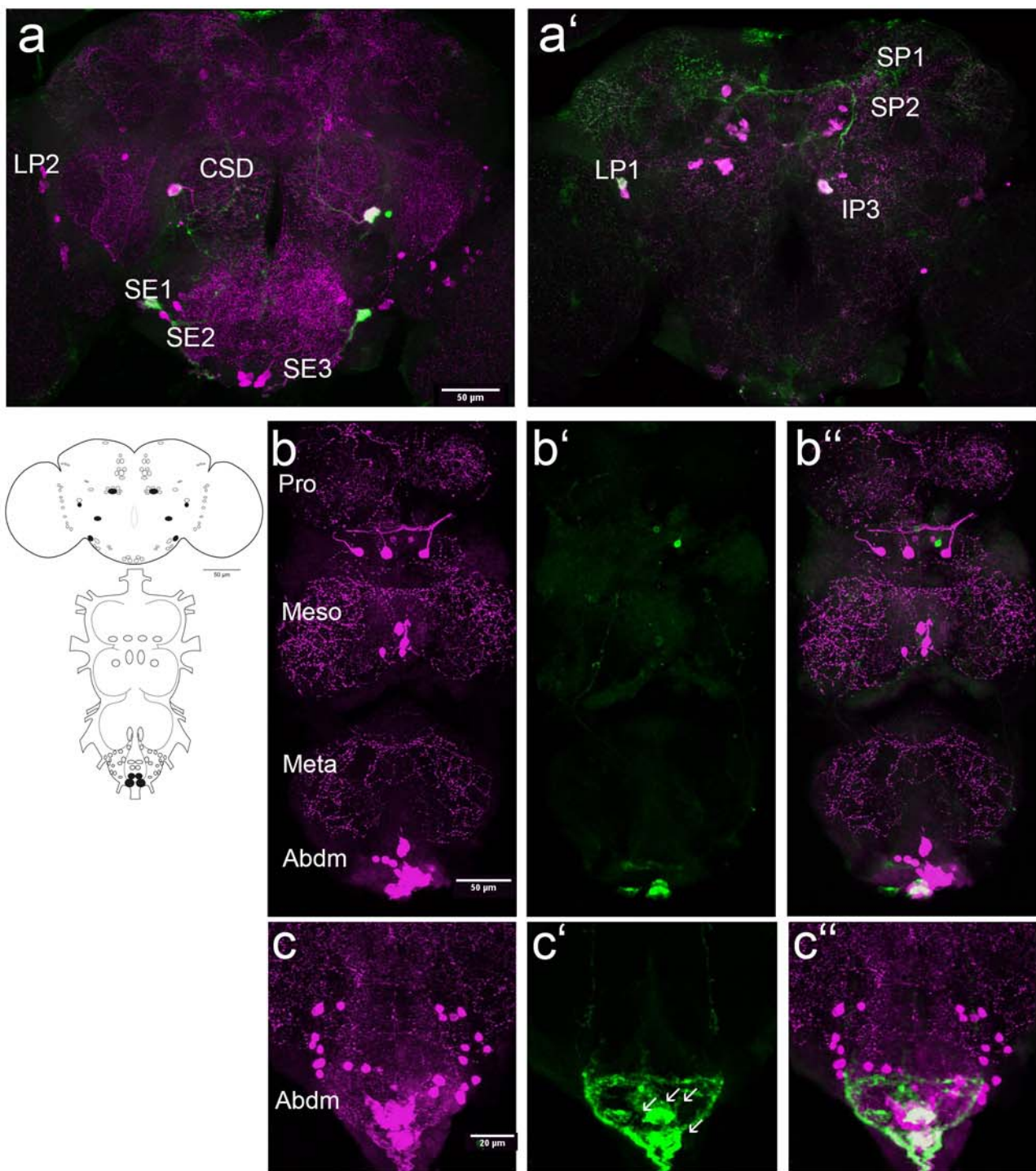

Figure-S4-Scholz

Supplement: S4 Fig — a–c, Serotonin IR is labelled in magenta, GFP is labelled in green, and co-expression of serotonin and GFP is shown in white. Images are merged in a, a′, b″to c″. a, Z-projection of the anterior and a′, posterior part of an adult male brain. b–b″, Pro-, meta- and mesothoracic segments with serotonergic neurons expressing Gal4. c–c″, A more detailed view of the abdomen (Abdm). The schematic summarizes the data presented in S1 Table; black circles indicate serotonergic neurons that express GFP, and empty circles indicate those that express serotonin only. (PDF) [file pone.0167518.s006.pdf]

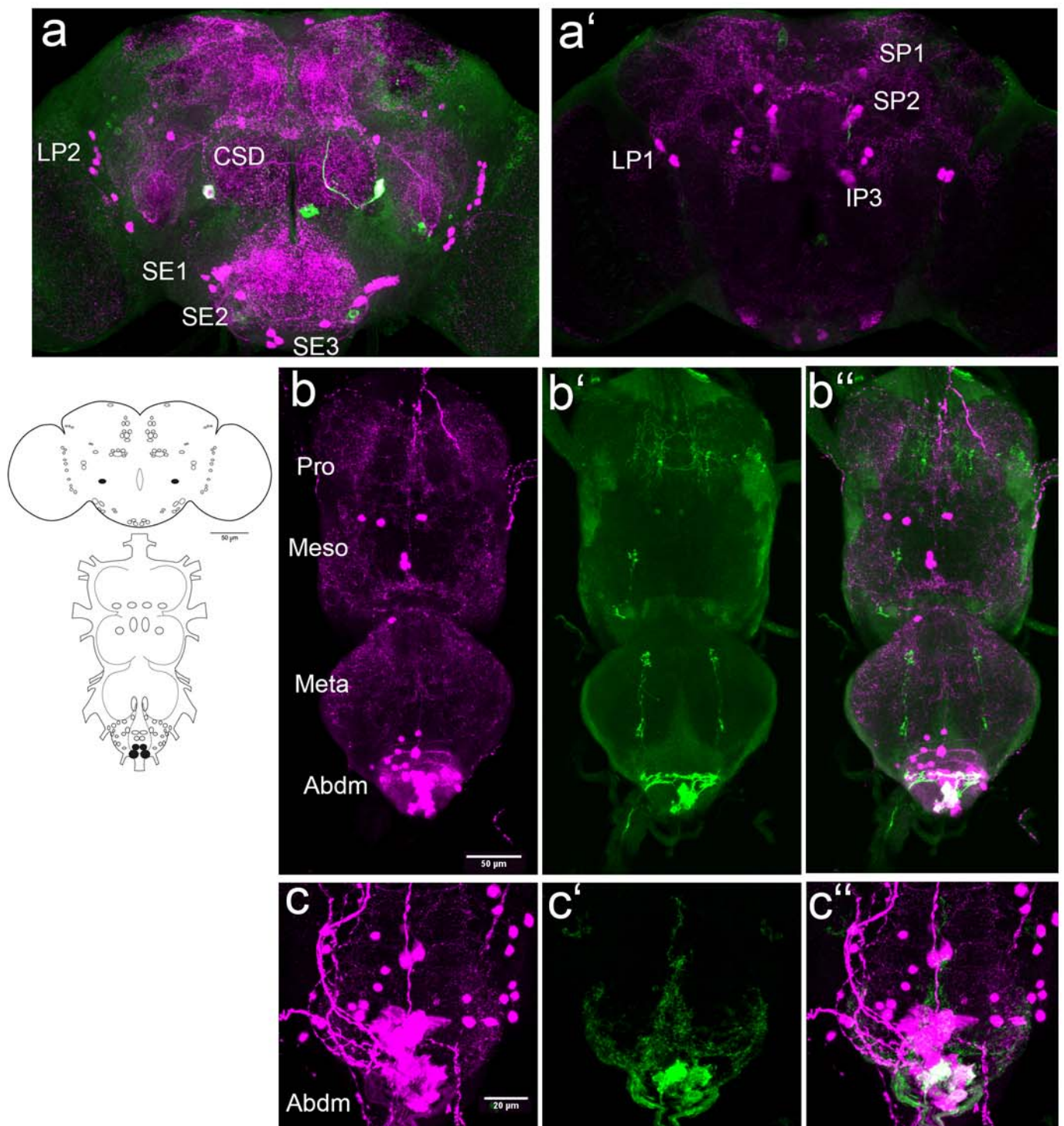

Figure-S5-Scholz

Supplement: S5 Fig — a–c, Serotonin IR is labelled in magenta, GFP is labelled in green, and co-expression of serotonin and GFP is shown in white. A merge is shown in a, a′, b″to c″. Z-projection of the a, anterior and a′, posterior part of an adult male brain. b–b″, Pro-, meta- and mesothoracic segments with serotonergic neurons expressing Gal4. A more detailed view of the abdomen (Abdm). The schematic summarizes the clusters expressing serotonin and GFP in black circles and serotonin only expressing cells with empty circles. (PDF) [file pone.0167518.s007.pdf]
